# Trends of Transmitted and Acquired Drug Resistance in Europe From 1981 to 2019: A Comparison Between the Populations of Late Presenters and Non-late Presenters

**DOI:** 10.3389/fmicb.2022.846943

**Published:** 2022-04-13

**Authors:** Mafalda N. S. Miranda, Marta Pingarilho, Victor Pimentel, Maria do Rosário O. Martins, Rolf Kaiser, Carole Seguin-Devaux, Roger Paredes, Maurizio Zazzi, Francesca Incardona, Ana B. Abecasis

**Affiliations:** ^1^Global Health and Tropical Medicine (GHTM), Institute of Hygiene and Tropical Medicine, New University of Lisbon (IHMT/UNL), Lisbon, Portugal; ^2^Institute of Virology, University of Cologne, Cologne, Germany; ^3^Laboratory of Retrovirology, Department of Infection and Immunity, Luxembourg Institute of Health, Esch-sur-Alzette, Luxembourg; ^4^Infectious Diseases Department and IrsiCaixa AIDS Research Institute, Hospital Universitari Germans Trias i Pujol, Badalona, Spain; ^5^Department of Medical Biotechnologies, University of Siena, Siena, Italy; ^6^IPRO—InformaPRO S.r.l., Rome, Italy; ^7^EuResist Network, Rome, Italy

**Keywords:** HIV-1 infection, transmitted drug resistance, acquired drug resistance, late presenters, non-late presenters

## Abstract

**Background:**

The increased use of antiretroviral therapy (ART) has decreased mortality and morbidity of HIV-1 infected people but increasing levels of HIV drug resistance threatens the success of ART regimens. Conversely, late presentation can impact treatment outcomes, health costs, and potential transmission of HIV.

**Objective:**

To describe the patterns of transmitted drug resistance (TDR) and acquired drug resistance (ADR) in HIV-1 infected patients followed in Europe, to compare its patterns in late presenters (LP) vs non-late presenters (NLP), and to analyze the most prevalent drug resistance mutations among HIV-1 subtypes.

**Methods:**

Our study included clinical, socio-demographic, and genotypic information from 26,973 HIV-1 infected patients from the EuResist Integrated Database (EIDB) between 1981 and 2019.

**Results:**

Among the 26,973 HIV-1 infected patients in the analysis, 11,581 (42.9%) were ART-naïve patients and 15,392 (57.1%) were ART-experienced. The median age was 37 (IQR: 27.0–45.0) years old and 72.6% were males. The main transmission route was through heterosexual contact (34.9%) and 81.7% of patients originated from Western Europe. 71.9% of patients were infected by subtype B and 54.8% of patients were classified as LP. The overall prevalence of TDR was 12.8% and presented an overall decreasing trend (*p* for trend < 0.001), the ADR prevalence was 68.5% also with a decreasing trend (*p* for trend < 0.001). For LP and NLP, the TDR prevalence was 12.3 and 12.6%, respectively, while for ADR, 69.9 and 68.2%, respectively. The most prevalent TDR drug resistance mutations, in both LP and NLP, were K103N/S, T215rev, T215FY, M184I/V, M41I/L, M46I/L, and L90M.

**Conclusion:**

Our study showed that the overall TDR (12.8%) and ADR (68.5%) presented decreasing trends during the study time period. For LP, the overall TDR was slightly lower than for NLP (12.3 vs 12.6%, respectively); while this pattern was opposite for ADR (LP slightly higher than NLP). We suggest that these differences, in the case of TDR, can be related to the dynamics of fixation of drug resistance mutations; and in the case of ADR with the more frequent therapeutic failure in LPs.

## Introduction

In 2014, UNAIDS implemented the Fast-Track approach driven by the 95-95-95 targets. These targets have the aim to end the pandemic by 2030 by achieving 95% of diagnosis among people living with HIV, 95% of those receiving antiretroviral treatment and 95% of those reaching viral suppression (Joint United Nations Programme on Hiv/Aids (Unaids), 2015). In the meantime, UNAIDS has developed a set of targets for 2025 to help achieve the previous goals until 2030, which are people-centered and right-based (Unaids).

At the end of 2020, there were 37.7 million people living with HIV and at least 50% of the new diagnoses were related to late HIV infection [late presenters (LP)], with regional differences. LP are patients newly diagnosed with a baseline CD4 count lower than 350 cells/mm^3^ or with an AIDS-defining event, regardless of CD4 cell count (Miranda et al., 2021). Between 2000 and 2020 the percentage of new HIV infections dropped by 49% and HIV-related deaths dropped by 55% due to antiretroviral therapy (ART; World Health Organization).

The advent of highly active ART has greatly improved the prognosis of HIV-1 infection and reduction of the risk of HIV transmission (Cdc). Today, 73% of people living with HIV have access to ART. Drug resistance could be acquired drug resistance (ADR), due to selective pressure of antiretrovirals (ARVs) in individuals, or transmitted drug resistance (TDR) due to an infection by HIV strains that harbor drug resistance mutations (DRMs; Clutter et al., 2016; Pingarilho et al., 2020).

Drug resistance testing is recommended for individuals with HIV infection who are newly diagnosed or ART-naïve patients, individuals on ART with a viral load higher than 200 copies/mL, individuals who did not achieve viral suppression, and individuals who interrupted ART with a non-nucleoside reverse transcriptase inhibitor (NNRTI; Günthard et al., 2019). For ART-naïve patients, genotypic drug-resistance testing involved testing for mutations in the reverse transcriptase (RT), protease (PR) and integrase (IN) genes. In ART-experienced patients, genotypic and phenotypic resistance testing is recommended in individuals suspect of multi drug-resistance mutations and virological failure (Nih).

The most common DRMs among ART-naïve and ART-experienced patients for nucleoside reverse transcriptase inhibitors (NRTIs) were M41L and M184V, respectively, and K103N for NNRTIs (Rossetti et al., 2018; Zou et al., 2020).

In 2016, the World Health Organization (WHO) recommended the following guidelines as a first-line ART regimen: the combination of two NRTIs, such as tenofovir (TDF) and lamivudine (3TC) or emtricitabine (FTC), plus an integrase strand inhibitor (INSTI), such as dolutegravir (DTG), or instead of DTG the combination with the NNRTI efavirenz (EFV). The recommendations for second-line regimens included the combination of two NRTIs plus one protease inhibitor (PI), like atazanavir (ATV) or lopinavir/ritonavir (LPV/RTV) or two NRTIs and DTG. Third-line regimens included the combination of one PI, such as darunavir (DRV), DTG, and one or two NRTIs (World Health Organization).

Resistance to ART could decrease the success of first line regimens and is a major threat to halt the UNAIDS targets, as well as late presentation. Resistance to antiretrovirals and late presentation are still existing problems that could delay the success of regimens and continue the onward transmission of HIV-1 infection. In this study, we aim to describe the patterns of TDR and ADR, as well as compare them in LP and non-late presenter (NLP) populations included in this study. We also analyzed the most prevalent drug resistance mutations and their prevalence in HIV-1 subtypes among LP and NLP HIV-1 infected patients followed in Europe.

## Methods

### Study Group

Clinical, socio-demographic, and genomic information from 26,973 HIV-1 infected patients from the EuResist Integrated Database (EIDB) between 1981 and 2019 were included in this study. The EIDB is one of the largest existing datasets which integrate clinical, socio-demographic, and viral genotypic information from HIV-1 patients. It integrates longitudinal, periodically updated data mainly from Italy (ARCA database), Germany (AREVIR database) Spain (CoRIS and IRSICAIXA), Sweden, Belgium, Portugal, and Luxembourg (EuResist; Lawyer et al., 2011; Zazzi et al., 2012).

In this study, information from the ARCA, AREVIR, Luxembourg, IRSICAIXA, and Portugal databases were used.

### Exclusion Criteria

Among the 89,851 HIV-1 infected patients included in the EuResist database, only 54,176 patients had sequence information for the RT and PR regions. Those patient sequences went through the quality control process. We calculated the ambiguity rate for each genomic sequence and only included those sequences that were larger than 500 nucleotides and with an ambiguity rate lower than 2.5%, resulting in the elimination of 4,044 sequences. Our final study population included 26,973 HIV-1 infected patients, because of the 50,132 patients, only 26,973 had information regarding their date of first ARV therapy.

### Institutional Review Board Statement

All procedures performed in this study were in accordance with the ethical standards of the institutional and/or national research committee and with the 1964 Helsinki declaration. The database enrolled anonymized patients’ information, including demographic, clinical, and genomic data from patients from the EuResist Integrated Database (Date of approval: January 15, 2021).

### Drug Resistance Analysis and Subtyping

HIV pol sequences were derived from existing routine clinical genotypic resistance tests (Sanger method, e.g., Viroseq, Trugene and in house genotyping). The size of RT and PR fragments used for this analysis were between 500 and 1,000 nucleotides. Only the first HIV genomic sequence per patient was analyzed. TDR was defined as the presence of one or more surveillance drug resistance mutations in a sequence, according to the WHO 2009 surveillance list (Bennett et al., 2009). The sequences were submitted to the Calibrated Population Resistance tool version 8.0. Clinical resistance to ARV drugs was calculated through the Standford HIVdb version 9.0.

We analyzed TDR and ADR overall proportions between 1981 and 2019, although we only used the years 1995–2019, divided into three time periods (1995–2002; 2003–2010, and 2011–2019), to compute TDR and ADR trends, since the absolute number before 1995 was smaller than 10 patients per year. We also analyzed TDR and ADR proportions in countries of follow-up. For this analysis, we limited the analyses to the last 10 years divided into two time periods (2008–2012 and 2013–2019).

HIV-1 subtyping was performed using the consensus of the result obtained based on three different subtyping tools: Rega HIV Subtyping Tool version 3.46^1^ (Pineda-Peña et al., 2013), COMET: adaptive context-based modeling for HIV-1^2^ (Struck et al., 2014) and SCUEAL.^3^

### Study Variables

New variables were created according to:

• Migrant/Native—based on country of origin and country of follow-up (if country of origin and country of follow-up is the same, then patient was classified as native; otherwise as migrant)
• Age at Drug Resistance Test—based on the difference between year of birth and date of the first drug resistance test;
• Region of Origin—based on country of origin;
• Treatment Status at Date of First Drug Resistance Test—based on the difference between sample collection date for first drug resistance test and start date of first therapy:

ART-naïve → patients who had a sample collection date for first drug resistance test before the start date of first therapy
ART-experienced → patients who had a sample collection date for first drug resistance test after the start date of first therapy

• Recentness of infection—based on ambiguity rate of genomic sequences. We defined as Chronic if the ambiguity rate was higher than 0.45% otherwise was defined as Recent infection, as previously described (Andersson et al., 2013).

LP vs NLP at HIV diagnosis- based on CD4 count, LP were defined as patients with a baseline CD4 count ≤ 350 cells/mm^3^ and NLP were defined as patients with baseline CD4 count > 350 cells/mm^3^ (Antinori et al., 2011).

### Statistical Analysis

The proportion and median [interquartile range (IQR)] were calculated for every categorical and continuous variable, respectively. The treatment status variable was compared with the categorical variables with the Chi-square test and continuous variables with the Mann-Whitney U test. Also, we analyzed the trends over time for the overall TDR and ADR through logistic regression models. Data was analyzed using RStudio (Version 1.2.5033).

## Results

### Characteristics of European Population

Among the 26,973 HIV-1 infected patients included in the analysis, 11,581 (42.9%) were ART-naïve patients and 15,392 (57.1%) were ART-experienced patients. Other socio-demographic characteristics of the population of patients has been analyzed and described in “Determinants of Determinants of HIV-1 Late Presentation in Patients Followed in Europe” (Miranda et al., 2021).

In the total population, the median age was 37 (IQR: 27.0–45.0) years old and 72.6% of HIV-1 infected patients were males. The main transmission route was through heterosexual contact (34.9%) and 81.7% were originated from Western Europe. The most prevalent subtype observed in this population was subtype B (71.9%). Most patients included in this study were native (77.4%) and as having chronic infection (63.6%) based on the ambiguity rate of the first genomic sequence. CD4 count at diagnosis and viral load at diagnosis (log10) presented a median of 318 cells/mm^3^ (IQR 151–513) and log10 4.3 copies/mL (IQR 3.3–5.0), respectively.

54.8% of patients were classified as LP (CD4 < 350 cells/mm^3^). In ART-naïve patients, 52.8% were LP, meanwhile in ART-experienced patients, 56.4% were LP at time of diagnosis (Table 1).

**TABLE 1:** Sociodemographic and clinical patient characteristics.

Patient characteristics	Total	ART-naive	ART-experienced	*p*-value
Total, *n* (%)	26973 (100)	11581 (42.9)	15392 (57.1)	
Gender, *n* (%)	26475 (98.2)	11458 (43.3)	15017 (56.7)	
Male	19224 (72.6)	8797 (76.8)	10427 (69.4)	*p* < 0.001
Female	7251 (27.4)	2661 (23.2)	4590 (30.6)	
Median age at resistance test in years IQR, *n* (%)	26973 (100)	11581 (42.9)	15392 (57.1)	*p* < 0.001
	37.0 (27.0–45.0)	37.0 (30.0–45.0)	37.0 (0.0–44.0)	
≤18	5047 (18.7)	761 (6.6)	4286 (27.8)	*p* < 0.001
19–30	3423 (12.7)	2468 (21.3)	955 (6.2)	
31–55	16707 (61.9)	7472 (64.5%)	9235 (60.0)	
≥56	1796 (6.7)	880 (7.6)	916 (6.0)	
Transmission route, *n* (%)	18118 (67.2)	8336 (46.0)	9782 (54.0)	
Heterosexual	6326 (34.9)	3130 (37.5)	3196 (32.7)	*p* < 0.001
MSM	6124 (33.8)	3863 (46.3)	2261 (23.1)	
IDU	4370 (24.1)	838 (10.1)	3532 (36.1)	
Other	1298 (7.2)	505 (6.1)	793 (8.1)	
Region of origin, *n* (%)	19881 (73.7)	9460 (47.6)	10421 (52.4)	
Western Europe	16249 (81.7)	7436 (78.6)	8813 (84.6)	*p* < 0.001
Eastern Europe	554 (2.8)	377 (4.0)	177 (1.7)	
Africa	2109 (10.6)	1051 (11.1)	1058 (10.2)	
South America	611 (3.1)	338 (3.6)	273 (2.6)	
Other	358 (1.8)	258 (2.7)	100 (1.0)	
Migrant status, *n* (%)	19881 (73.7)	9460 (47.6)	10421 (52.4)	
Migrant	4494 (22.6)	2616 (27.7)	1878 (18.0)	*p* < 0.001
Native	15387 (77.4)	6844 (72.3)	8543 (82.0)	
Recentness of infection, *n* (%)	26973 (100)	11581 (42.9)	15392 (57.1)	
Chronic	17151 (63.6)	6915 (59.7)	10236 (66.5)	*p* < 0.001
Recent	9822 (36.4)	4666 (40.3)	5156 (33.5)	
Subtype, *n* (%)	26973 (100)	11581 (42.9)	15392 (57.1)	
HIV-1 Subtype B	19387 (71.9)	8047 (69.5)	11340 (73.7)	*p* < 0.001
HIV-1 Subtype non-B	7586 (28.1)	3534 (30.5)	4052 (26.3)	
Median (IQR) CD4 count at diagnosis (cells/mL), *n* (%)	24442 (90.6)	10937 (44.7)	13505 (55.3)	*p* < 0.001
	318.0 (151.0–513.0)	332.0 (160.0–518.0)	306.0 (147.0–508.5)	
LP	13390 (54.8)	5776 (52.8)	7614 (56.4)	*p* < 0.001
NLP	11052 (45.2)	5161 (47.2)	5891 (43.6)	
Viral Load at diagnosis (log10 copies/mL), *n* (%), IQR	14005 (51.9)	4589 (32.8)	9416 (67.2)	*p* < 0.001
	4.3 (3.3–5.0)	4.6 (3.8–5.3)	4.1 (3.2–4.9)	
≤4.0	5814 (41.5)	1410 (30.7)	4404 (46.8)	*p* < 0.001
4.1–5.0	4573 (32.7)	1580 (34.4)	2993 (31.8)	
≥5.1	3618 (25.8)	1599 (34.8)	2019 (21.4)	

### Transmitted and Acquired Drug Resistance

The overall prevalence of TDR was 12.8% (95%CI: 12.2–13.4%). NRTI, NNRTI and PI TDR were detected in 8.2% (95%CI: 7.7–8.7%), 5.6% (95%CI: 5.2–6.0%) and 3.7% (95%CI: 3.4–4.1%) of ART-naïve patients, respectively. 9.1% (95%CI: 8.6–9.7%) of these patients presented single class resistance, 2.8% (95%CI: 2.5–3.1%) presented dual class resistance and 0.9% (95%CI: 0.8–1.1%) presented triple class resistance (Table 2).

**TABLE 2:** Proportion of transmitted drug (TDR) and acquired drug resistance (ADR) between 1991 and 2019.

	Transmitted drug resistance (TDR)			Acquired drug resistance (ADR)		
		
	*n* (%)	95% CI	*p* for trend	*n* (%)	95% CI	*p* for trend
Total	11581 (100)			15392 (100)		
Any DRMs	1482 (12.8)	12.2–13.4	<0.001	10543 (68.5)	67.8–69.2	<0.001
NRTI resistance	944 (8.2)	7.7–8.7	<0.001	9089 (59.1)	58.3–59.8	<0.001
NNRTI resistance	644 (5.6)	5.2–6.0	<0.001	6499 (42.2)	41.4–43.0	<0.001
PI resistance	427 (3.7)	3.4–4.1	<0.001	3727 (24.2)	23.5–24.9	<0.001
Single class resistance	1056 (9.1)	8.6–9.7	0.049	3617 (23.5)	22.8–24.2	<0.001
Dual class resistance	319 (2.8)	2.5–3.1	<0.001	5080 (33.0)	32.3–33.8	<0.001
Triple class resistance	107 (0.9)	0.8–1.1	<0.001	1846 (12.0)	11.5–12.5	<0.001
PI + NRTI resistance	115 (1.0)	0.8–1.2	<0.001	1671 (10.9)	10.4–11.4	<0.001
PI + NNRTI resistance	13 (0.1)	0.07–0.2	0.452	63 (0.4)	0.3–0.5	0.179
NRTI + NNRTI resistance	191 (1.6)	1.4–1.9	<0.001	3346 (21.7)	21.1–22.4	<0.001

*p-value for trend of TDR and ADR between 1995 and 2019. DRM, drug resistance mutations; NRTI, nucleotide reverse transcriptase inhibitors; NNRTI, non-nucleotide reverse transcriptase inhibitors; PI, protease inhibitors; CI, confidence interval.*

68.5% (95%CI: 67.8–69.2%) of experienced patients presented ADR, with higher drug resistance mutations for NRTI (59.1%; 95%CI: 58.3–59.8%), followed by NNRTI (42.2%; 95%CI: 41.4–43.0%) and by PI (24.2%; 95%CI: 23.5–24.9%). 23.5% (95%CI: 22.8–24.2%) of ART-experienced patients presented single class resistance, 33.0% (95%CI: 32.3–33.8%) presented dual class resistance and 12.0% (95%CI: 11.5–12.5%) presented triple class resistance (Table 2).

**FIGURE 1:**
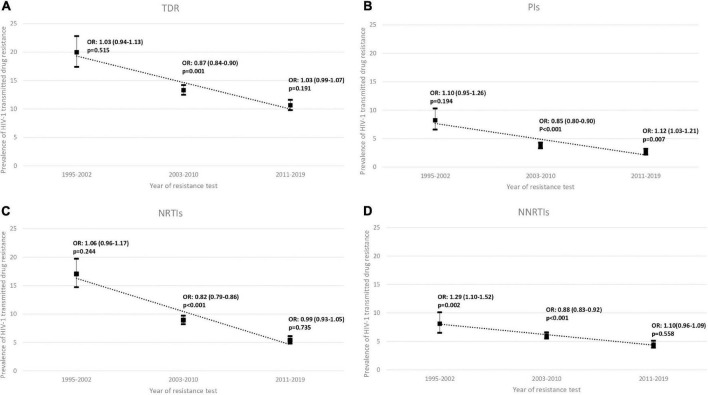
Proportion of **(A)** overall transmitted drug resistance (TDR), **(B)** of protease inhibitors (PIs), **(C)** of nucleoside reverse transcriptase inhibitor (NRTIs) and **(D)** of non-nucleoside reverse transcriptase inhibitor (NNRTIs) in sequences from drug-naïve patients between three periods 1995–2002, 2003–2010, and 2011–2019. OR, Odds Ratio; *p*, *p*-value.

TDR presented an overall decreasing trend between 1995 and 2019 (*p* for trend < 0.001; Table 2 and Supplementary Data). The same decreasing trend for TDR was observed for NRTIs, NNRTIs and PIs drug classes (*p* for trend < 0.001; Table 2). TDR between three time-periods (1995–2002; 2003–2010, and 2011–2019) was analyzed and it was observed that the overall TDR decreased from 20.0% to 13.3% to 10.7%. The same happened for every drug class, PIs (8.2% to 3.8% to 2.7% for the three time-periods, respectively), NRTIs (17.0% to 8.9% to 5.4% for the three time-periods, respectively) and for the NNRTIs (8.1% to 6.0% to 4.4% for the three time-periods, respectively). Moreover, between the 2003–2010 time-period, the overall TDR had a statistically significant decreasing trend (OR = 0.87; *p* = 0.001; Figure 1A). For the same time-period, the ARV drug classes also showed a decreasing trend, PI (OR = 0.85; *p* < 0.001), NNRTIs (OR = 0.82; *p* < 0.001) and NNRTIs (OR = 0.88; *p* < 0.001; Figures 1A–D).

**FIGURE 2:**
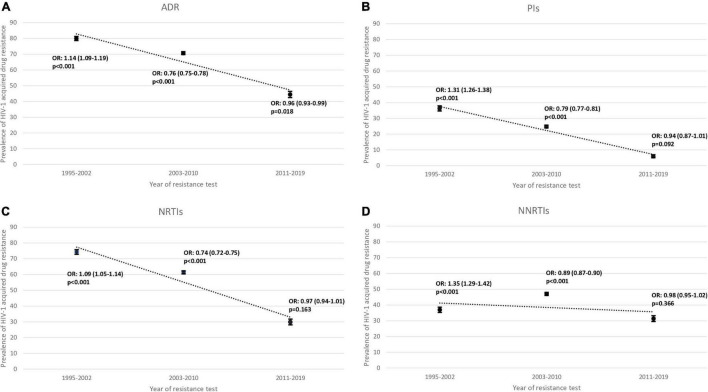
Proportion of **(A)** overall acquired drug resistance (ADR), **(B)** of protease inhibitors (PIs), **(C)** of nucleoside reverse transcriptase inhibitor (NRTIs) and **(D)** of non-nucleoside reverse transcriptase inhibitor (NNRTIs) in sequences from drug-experienced patients between three periods 1995–2002, 2003–2010 and 2011–2019. OR, Odds Ratio; *p*, *p*-value.

Regarding the overall ADR trend, it has been decreasing over the three time-periods (80.0% to 70.7% to 44.5%) as well as in all drug classes studied except for NNRTIs (Figure 1A). PIs decreased from 36.3% to 24.8% to 5.9% and NRTIs decreased from 74.3% to 61.4% to 29.8%. Conversely, NNRTIs increased from 36.9% to 47.0% and then decreased to 31.4%. In the last time-period, 2011–2019, the overall ADR showed a decreasing trend (OR = 0.96; *p* = 0.018). The drug classes, in the same time-period, also showed a decreasing trend, but without being statistically significant PIs (OR = 0.94; *p* = 0.092), NRTIs (OR = 0.97; *p* = 0.163) and NNRTIs (OR = 0.98; *p* = 366; Figure 2A–D).

**FIGURE 3:**
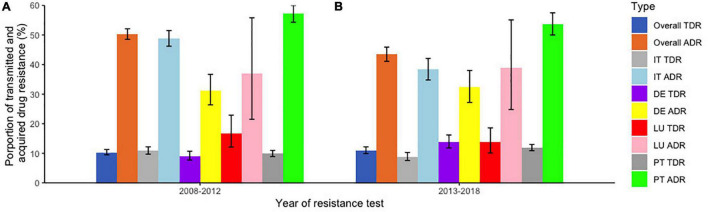
Proportion of transmitted and acquired drug resistance per country of follow-up in two different time periods. **(A)** Between 2008 and 2012; **(B)** between 2013 and 2018. TDR, Transmitted drug resistance; ADR, Acquired drug resistance; IT, Italy; DE, Germany; LU, Luxembourg; PT, Portugal.

Differences in TDR and ADR prevalence between different countries included in this study were also analyzed between two time-periods (2008–2012 and 2013–2018). In our study population, in the first time-period (2008–2012), Luxembourg had the higher rate of TDR (16.8%). This scenario changed for TDR when the last time-period (2013–2018) was analyzed, since Germany (13.9%) presented the highest TDR rate. Comparing each country in those two time-periods, the TDR rate of Italy and Luxembourg decreased from one period to another (10.9% to 8.8%; 16.8% to 13.8%, respectively), while the rates of Germany and Portugal increased (9.9% to 11.9%; 9.1% to 13.9%, respectively). The ADR rates for the first time-period, indicated that all the countries, with the exception of Portugal (57.2%), presented a ADR lower than 50% (Figure 3A) and for the last time-period Portugal maintained the highest rate (53.7%; Figure 3B). Comparing the ADR rates between the same time-periods, the rate of Italy and Portugal decreased from one period to another (48.9% to 38.4%; 57.2% to 53.7%, respectively), while the rates of Germany and Luxembourg increased (31.3% to 32.4%; 37% to 38.9%, respectively; Figure 3).

### Transmitted and Acquired Drug Resistance Among Late Presenters and Non-late Presenters

Focusing now on the LP and NLP population, we observed a TDR of 12.3% (95%CI: 11.5–13.2) for LP population and 12.6% (95%CI: 11.8–13.6) for NLP population. In relation to drug resistance classes, the rates of resistance were higher in the NLP when compared to LPs, except for the NNRTIs class. LP presented higher rates of ADR—69.9% (95%CI: 68.8–70.9)—when compared to NLP: 68.2% (95%CI: 67.0–69.3). Contrary to TDR, the rates of ADR were higher in LP when compared to NLP (Table 3).

**TABLE 3:** Proportion of transmitted drug (TDR) and acquired drug resistance (ADR) in Late Presenters (LP) and Non-Late Presenters (NLP) between 1991 and 2019.

Transmitted drug resistance (TDR)	Late presenters (LP)		Non-late presenters (NLP)	
		
	*n* (%)	95% CI	*n* (%)	95% CI
Total	5776 (100)		5161 (100)	
Any DRMs	710 (12.3)	11.5–13.2	652 (12.6)	11.8–13.6
NRTI resistance	446 (7.7)	7.1–8.4	428 (8.3)	7.6–9.1
NNRTI resistance	317 (5.5)	4.9–6.1	269 (5.2)	4.6–5.9
PI resistance	202 (3.5)	3.1–4.0	191 (3.7)	3.2–4.3
**Acquired drug resistance (ADR)**				
Total	7614 (100)		5891 (100)	
Any DRMs	5319 (69.9)	68.8–70.9	4016 (68.2)	67.0–69.3
NRTI resistance	4588 (60.3)	59.2–61.4	3538 (60.1)	58.6–61.1
NNRTI resistance	3354 (44.1)	42.9–45.2	2327 (39.5)	38.3–40.8
PI resistance	2047 (26.9)	25.9–27.9	1328 (22.5)	21.5–23.6

*DRM, drug resistance mutations; NRTI, nucleotide reverse transcriptase inhibitors; NNRTI, non-nucleotide reverse transcriptase inhibitors; PI, protease inhibitors; CI, confidence interval.*

**FIGURE 4:**
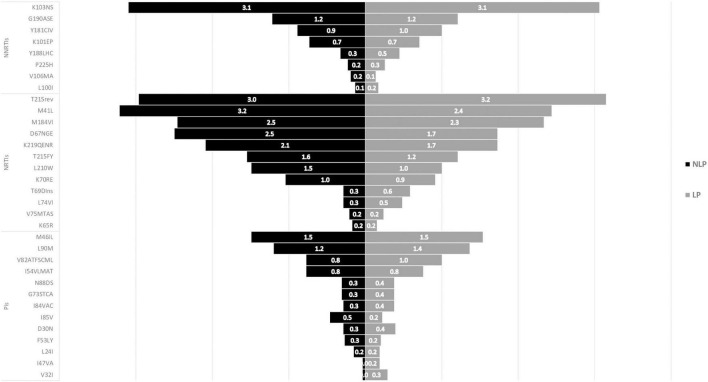
ART-naïve mutations in Non-Late-Presenters (NLP) vs Late Presenters (LP). PIs, protease inhibitors; NRTIs, nucleoside reverse transcriptase inhibitor; NNRTIs, non-nucleoside reverse transcriptase inhibitor.

In both LP and NLP populations, the NNRTIs class K103N/S mutation presented the highest prevalence (3.1%; Figure 4). For PIs, M46I/L was more prevalent (1.5% for both LP and NLP) followed by L90M (1.4% for LP and 1.2% for NLP). Futhermore, in the PIs class there were two mutations present in LP (I47VA and V32I, respectively), that were not present in NLP (Figure 4). In the NLP, for NRTIs, we observed that M41I/L (3.2%) was the mutation with highest prevalence, followed by T215 revertants (3.0%) and by D67N/G/E and M184I/V (2.5%). Conversely, in the LP population, T215 revertants were more prevalent (3.2%), followed by M41I/L (2.4%) and M184I/V (2.3%).

**FIGURE 5:**
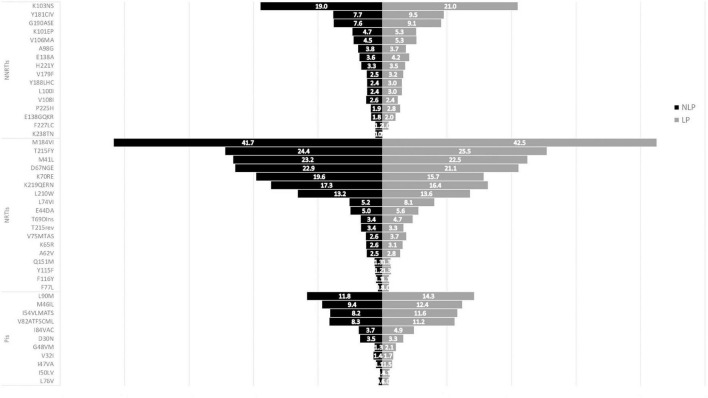
ART-experienced mutations in Non-Late-Presenters (NLP) vs Late Presenters (LP). PIs, protease inhibitors; NRTIs, nucleoside reverse transcriptase inhibitor; NNRTIs, non-nucleoside reverse transcriptase inhibitor.

Drug resistance mutations in ART-experienced patients in both LP and NLP populations were also analysed and compared (Figure 5). The more prevalent mutations consistently presented higher prevalences in LPs than in NLPs. Similarly to ART-naïve patients, for NNRTIs drug class, K103N/S mutation presented the highest prevalence (21.0% in LP and 19.0%, in NLP; Figure 5). For NRTIs, M184I/V had the highest prevalence (42.5% for LP and 41.7% for NLP). In the PIs class, the mutations with higher prevalence were L90M (11.8% NLP and 14.3% LP) and M46I/L (9.4% for NLP and 12.4% for LP). Also, K238TN mutation from the NNRTIs class was present only in the LP population. The presence of these mutations could lead to reduced susceptibility to some specific ARV.

### Analysis of Mutations Per Subtype Among Late Presenters and Non-late Presenters Patients

**FIGURE 6:**
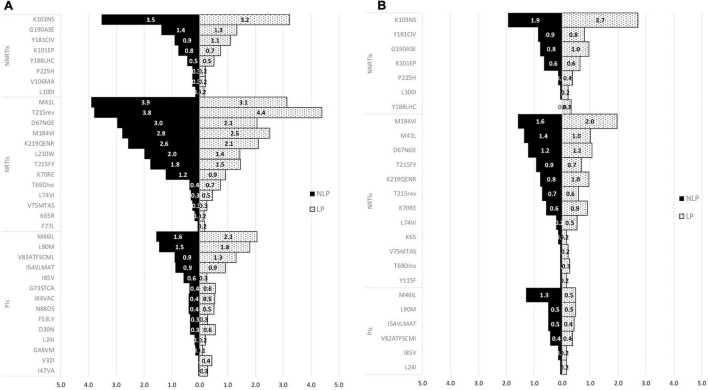
Mutations in Non-Late presenters (NLP) and Late presenters (LP) in subtype B **(A)** and subtype non-B **(B)** for ART-naïve patients. PIs, protease inhibitors; NRTIs, nucleoside reverse transcriptase inhibitor; NNRTIs, non-nucleoside reverse transcriptase inhibitor.

Finally, we compared mutations in LP and NLP, according to subtype B and non-B subtypes. As we can see in Figure 6, for subtype B ART-naïve patients, for both NRTIs and NNRTIs, most mutations—except T215rev—were more prevalent in NLP when compared to LP. K103N/S mutation was the one with higher prevalence for NNRTIs (3.5% for NLP and 3.2% for LP). For NRTIs, M41L was the mutation with highest prevalence (3.9% for NLP vs 3.1% for LP), while for LP it was T215rev mutation (4.4% LP vs 3.8% NLP). For the PIs class, conversely, M46I/L and L90M were the mutations with the highest prevalence with higher prevalence in LP compared to NLP (1.6 and 1.5% for NLP and 2.1 and 1.8% for LP, respectively; Figures 6A,B).

Regarding the non-B subtypes, K103N/S mutation was more prevalent in LP compared to NLP (2.7 vs 1.9%, respectively) which was the one with the highest prevalence. For NRTIs, M184V/I, M41L and D67NGE mutations (1.6, 1.4, and 1.2% for NLP and 2.0, 1.0, and 1.1 for LP, respectively) were the ones with higher prevalence. For PIs, M46I/L (1.3% for NLP and 0.5% for LP) was the one with the higher prevalence (Figures 6A,B). Comparing both populations regarding subtype non-B, opposite to what happens in subtype B, we observed that the LP population carried higher a prevalence of the most prevalent mutations (Figures 6A,B). Also, the K103N/S and the M184V/I were the mutations that were present in more non-B subtypes in the LP population, while the M46I/L was the one for the NLP populations. The most prevalent non-B subtype was subtype C (data not shown).

**FIGURE 7:**
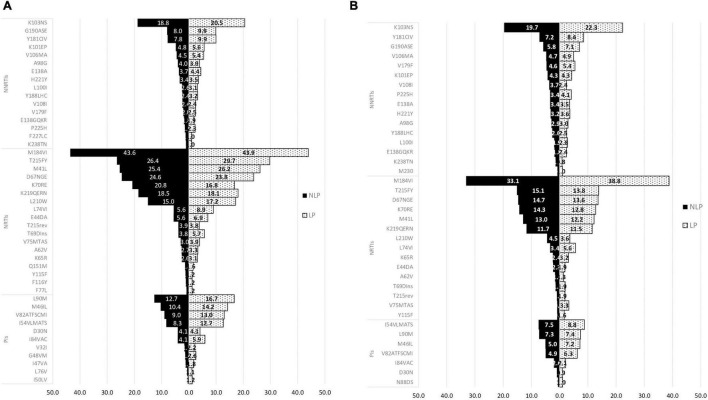
Mutations in Non-Late presenters (NLP) and Late presenters (LP) in subtype B **(A)** and subtype non-B **(B)** for ART-experienced patients. PIs, protease inhibitors; NRTIs, nucleoside reverse transcriptase inhibitor; NNRTIs, non-nucleoside reverse transcriptase inhibitor.

In ART-experienced patients, both in subtype B and in non-B subtypes, the most prevalent mutations occurred more frequently in LP than in NLP. For NNRTIs class K103N/S mutation had the highest prevalence in both NLP and LP (18.8 and 20.5%, respectively). For NRTIs the mutation with the highest prevalence was M184V/I mutation (43.6% for NLP and 43.9% for LP), and for PIs L90M and M46I/L were the mutations with the highest prevalence (12.7 and 10.4% for NLP and 16.7 and 14.2% for LP, respectively; Figures 7A,B).

Regarding the non-B subtypes, similiar to subtype B, K103N/S mutation (19.7% for NLP and 22.3% for LP) for NNRTIs, and M184I/V (33.1% for NLP and 38.8% LP) for NRTIs, were the ones with the highest prevalence. While in the PIs class, I54VLMATS (7.5% for NLP and 8.8% for LP) and L90M mutations (7.3% for NLP and 7.4% for LP) were the ones with the higher prevalence (Figures 7A,B). Also, M184V/I was the mutation that was present in the most diversity and proportion of non-B subtypes in both NLP and LP populations. The most prevalent non-B subtype was subtype G (data not shown).

## Discussion

There are no recent studies with updated information regarding TDR and ADR prevalence in Europe and the most recent study about this topic only includes TDR and is based on the median overall values from different studies (Rhee et al., 2020). In our study, we presented updated information of the prevalence of TDR and ADR in the overall population and compared its patterns between LP and NLP. Overall, TDR had a prevalence of 12.8% and ADR of 68.5%. The TDR and ADR prevalence from our study was slightly higher when compared to other studies and this could be explained by the fact that our timeline includes patients diagnosed between 1981 and 2019 (Tostevin et al., 2017; Zazzi et al., 2018). Regarding the overall trends, both TDR and ADR presented a decreasing trend, consistently with other studies in and outside of Europe (Schmidt et al., 2014; Rocheleau et al., 2018).

We also compared TDR and ADR for the countries of follow-up included in the database divided into two time periods (2008–2012 and 2013–2018). For Italy, TDR prevalence decreased within time-periods (2008–2012:10.9% and 2013–2018: 8.8%), which is in accordance with studies from that country and around the same timeline (Franzetti et al., 2018; Rossetti et al., 2018). The prevalence of ADR also decreased in Italy (2008–2012: 48.9% and 2013–2018; 38.4%), and these results are slightly lower than those from a study from the Italian ARCA database. Moreover, the decrease in the last 5 years is in accordance with that study (Lombardi et al., 2021). For Germany, TDR prevalence was 9.1% and ADR prevalence was 31.3% between 2008 and 2012, and for a similar time-period, the TDR rate was around the same, but our ADR rate was lower than in another study reported in this country (Schmidt et al., 2014). For Luxembourg, the TDR prevalence was 16.8% and the ADR prevalence was 37% between 2008 and 2012, which is higher when compared to the values in Europe (Hofstra et al., 2016). For Portugal, TDR prevalence increased between time-periods (2008–2012:9.9% and 2013–2018: 11.9%), while ADR prevalence decreased between the same time-periods (2008–2012: 57.2% and 2013–2018: 53.7%). The TDR prevalence in the first time-period was closer to the one from a study conducted in Portugal between 2001 and 2017 and that same study indicated an increase trend for TDR. Our ADR prevalence for Portugal in the first time-period, had a lower value than the overall ADR prevalence from that study, although the decreasing trend was concordant (Pingarilho et al., 2020).

We also compared drug resistance in LP vs NLP, both in ART-naïve or ART-experienced patients. There were no major differences in the prevalence of drug resistance mutations in both LP and NLP from the ART-naïve population. However, LPs presented a lower prevalence of TDR than NLP, potentially suggesting a reversion of these mutations when patients are diagnosed late. The most prevalent mutations were the K103N/S, T215 revertants, the M184V/I, the M41I/L, the M46I/L and the L90M. However, in the LP, there were two mutations—I47V/A and V32I—that were not present in the NLP. Despite the lack of significance of these findings, we were not expecting to find mutations occurring specifically in late presenters, that could eventually indicate the irreversible fixation of these mutations in some cases, where they are not associated with a fitness cost (Winand et al., 2015; Nagaraja et al., 2016). In the ART-experienced population, there were also no significant differences between the LP and NLP populations, however, LPs presented a higher prevalence of ADR compared to NLP. The most prevalent mutations among LP and NLP were the K103N/S, the M184IV/I, the L90M and M46I/L. The K103N/S mutation presented similar prevalence in LP and NLP in ART-naïve, while ART-experienced LP had higher prevalence compared to NLP (Hiv Drug Resistance Database). T215rev in drug naïve patients was more prevalent in LP compared to NLP. The NRTIs T215rev mutants is associated with risk of virological failure to zidovidine (AZT) or stavudine (d4T). M41I/L impacts negatively virological response to regimens with abacavir (ABC), didanosine (ddl) or tenofovir (TDF). Together, these mutations confer high-level resistance to AZT and d4T. For the same drug class, M184V/I mutation reduces susceptibility to lamivudine (3TC) and emtricitabine (FTC; Hiv Drug Resistance Database). PI mutations were consistently more prevalent in LP compared to NLP, both in experienced and naïve patients, indicating a potential irreversible fixation of these mutations when they occur. The most prevalent were M46I/L which is associated with a reduction in the susceptibility to atazanavir (ATV), fosamprenavir (FPV), indinavir (IDV), lopinavir (LPV) and NFV, and L90M which is associated to reduced susceptibility to almost all PIs, except for tipranavir (TPV) and darunavir (DRV; Hiv Drug Resistance Database).

It is known that some mutations are closely related to specific subtypes and recombinant forms. As such, we conducted a final analysis distinguishing the patterns found in subtype B when compared to non-B subtypes. The most prevalent subtype was subtype B and the mutation with the highest prevalence in NLP ART-naïve patients was M41L from the NRTIs drug class. This result is in accordance with a study of mutations according to subtypes in Brazil (Westin et al., 2011).

In the LP and NLP patients, in the ART-experienced population, for both subtypes B and non-B, M184V/I mutation was the one with the higher prevalence.

This study was the first to analyze and compare transmitted and ADR in LP and NLP populations. Despite the lack of significant differences, we consistently found higher levels of TDR in NLP and higher levels of ADR in LP. We find this pattern consistent, except for non-B subtypes and the PIs class. This suggests different dynamics of reversion and irreversible fixation of mutations that should be further investigated in future studies.

### Limitations

Our study had some limitations. For example, concerning the analysis time-period, the first years and the more recent ones can be a bias in the analysis, since the number of individuals of those years is low compared to other years of resistance test collection date. Also, our population is mainly from Western Europe, providing a certain imbalance when characterizing the population and the TDR and ADR origins regarding geographical distribution. Another limitation of our study is the definition of LP as there is lack of consensus as to whether this definition (“baseline CD4 count in newly diagnosed patient is lower than 350 cells/mm^3^ or has an AIDS-defining event, regardless of CD4 cell count”) is the correct one to characterize those who present late to diagnosis. Some discuss that the threshold should be CD4 count lower than 200 cells/mm^3^, i.e., those characterized in LP with advanced disease.

## Conclusion

In conclusion, our study showed that the overall TDR and ADR had a decreasing trend and the prevalence has been steady through the years. There were no significant differences in the TDR rate between the LP and NLP (around 12% in both), with slightly higher levels in the NLP. The mutation profile was also similar, again with most mutations presenting a higher prevalence of TDR in NLP and higher prevalence of ADR in LP. Late presentation for HIV remains a key unresolved challenge in HIV/AIDS with serious adverse consequences at the individual and societal levels. Our study highlights ADR and TDR patterns and drug resistance mutations, alone and according to subtypes in the LP population, when compared to NLP.

## Data Availability Statement

The original contributions presented in the study are included in the article/Supplementary Material, further inquiries can be directed to the corresponding author/s.

## Author Contributions

MNSM, MP, and AA: conceptualization. MNSM, MP, VP, MdROM, and AA: methodology. MNSM, VP: software. MNSM, MP, FI, and AA: validation. MNSM, VP, MP, and AA: formal analysis. MNSM, MP, VP, and MdROM: investigation. CS-D, RP, RK, MZ, and FI: resources. CS-D, RP, RK, MZ, and FI: data curation. MNSM, MP, and AA: writing—original draft preparation. MNSM, MP, FI, and AA: writing—review, and editing. MNSM, MP, VP, MdROM, and AA: visualization. AA: supervision, project administration, and funding acquisition. All authors contributed to the article and approved the submitted version.

## Conflict of Interest

FI was employed by IPRO—InformaPRO S.r.l. The remaining authors declare that the research was conducted in the absence of any commercial or financial relationships that could be construed as a potential conflict of interest. The authors declare that this study received funding from Gilead Sciences. The funder was not involved in the study design, collection, analysis, interpretation of data, the writing of this article, or the decision to submit it for publication.

## Publisher’s Note

All claims expressed in this article are solely those of the authors and do not necessarily represent those of their affiliated organizations, or those of the publisher, the editors and the reviewers. Any product that may be evaluated in this article, or claim that may be made by its manufacturer, is not guaranteed or endorsed by the publisher.

## Footnotes

1
https://www.genomedetective.com/app/typingtool/hiv

2
https://comet.lih.lu

3
http://classic.datamonkey.org/dataupload_scueal.php
