# Supplementary material for: Trends of Transmitted and Acquired Drug Resistance in Europe From 1981 to 2019: A Comparison Between the Populations of Late Presenters and Non-late Presenters
Source: Front Microbiol. 2022 Apr 13;13:846943. doi: 10.3389/fmicb.2022.846943 (PMC9044068; doi:10.3389/fmicb.2022.846943)
Supplement: Supplementary file 1 [file Data_Sheet_1.docx]

**Supplementary data:**

**
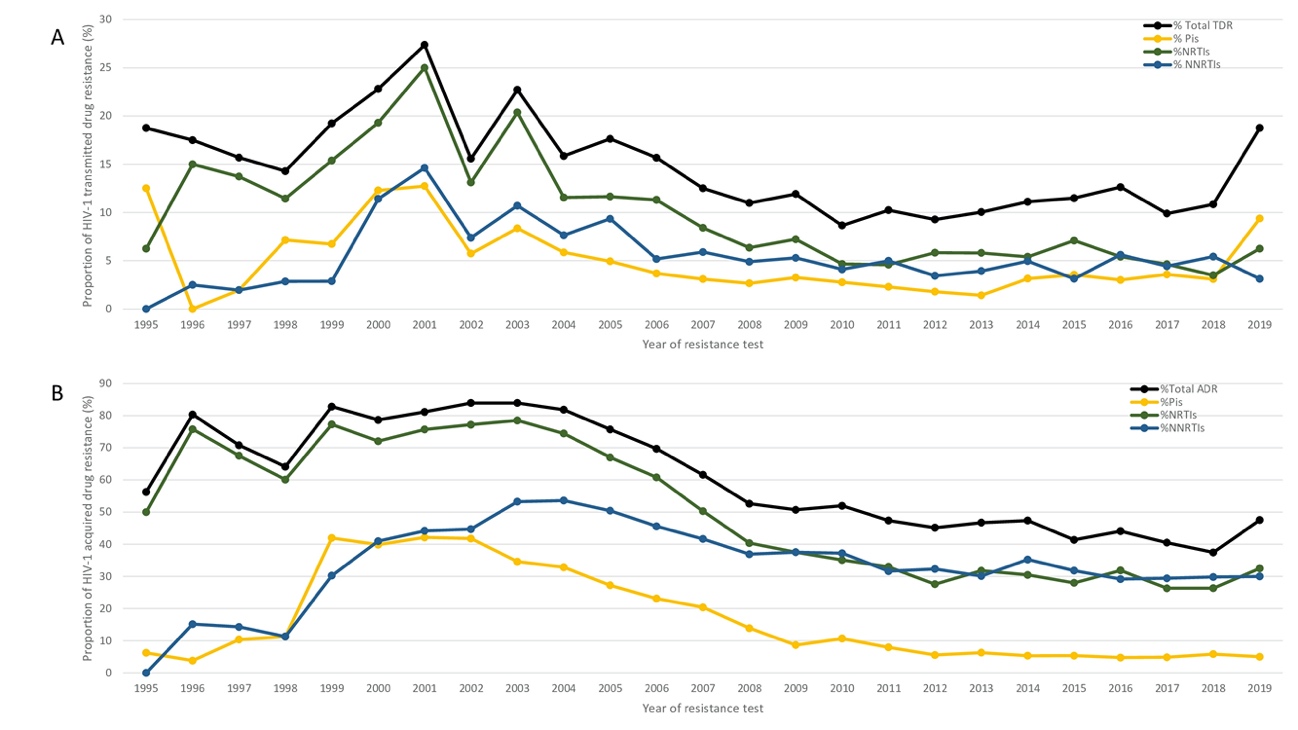
**

**S1.** Proportion of (A) transmitted drug resistance (TDR) in sequences from drug-naïve patients between 1995 and 2019 and (B) of acquired drug resistance (ADR) in drug-experienced patients between 1995 and 2019. NRTI- nucleotide reverse transcriptase inhibitors; NNRTI- non-nucleotide reverse transcriptase inhibitors; PI- protease inhibitors.
